# Ototoxicity: a high risk to auditory function that needs to be monitored in drug development

**DOI:** 10.3389/fnmol.2024.1379743

**Published:** 2024-05-02

**Authors:** Marie-Pierre Pasdelou, Lise Byelyayeva, Susanna Malmström, Sylvie Pucheu, Marie Peytavy, Hugo Laullier, Donald B. Hodges, Abraham R. Tzafriri, Gaëlle Naert

**Affiliations:** ^1^Cilcare, Montpellier, France; ^2^CBSET, Lexington, MA, United States

**Keywords:** ototoxicity, side effects, hearing monitoring, regulatory, guidance, drug development

## Abstract

Hearing loss constitutes a major global health concern impacting approximately 1.5 billion people worldwide. Its incidence is undergoing a substantial surge with some projecting that by 2050, a quarter of the global population will experience varying degrees of hearing deficiency. Environmental factors such as aging, exposure to loud noise, and the intake of ototoxic medications are implicated in the onset of acquired hearing loss. Ototoxicity resulting in inner ear damage is a leading cause of acquired hearing loss worldwide. This could be minimized or avoided by early testing of hearing functions in the preclinical phase of drug development. While the assessment of ototoxicity is well defined for drug candidates in the hearing field – required for drugs that are administered by the otic route and expected to reach the middle or inner ear during clinical use – ototoxicity testing is not required for all other therapeutic areas. Unfortunately, this has resulted in more than 200 ototoxic marketed medications. The aim of this publication is to raise awareness of drug-induced ototoxicity and to formulate some recommendations based on available guidelines and own experience. Ototoxicity testing programs should be adapted to the type of therapy, its indication (targeting the ear or part of other medications classes being potentially ototoxic), and the number of assets to test. For multiple molecules and/or multiple doses, screening options are available: *in vitro* (otic cell assays), *ex vivo* (cochlear explant), and *in vivo* (in zebrafish). In assessing the ototoxicity of a candidate drug, it is good practice to compare its ototoxicity to that of a well-known control drug of a similar class. Screening assays provide a streamlined and rapid method to know whether a drug is generally safe for inner ear structures. Mammalian animal models provide a more detailed characterization of drug ototoxicity, with a possibility to localize and quantify the damage using functional, behavioral, and morphological read-outs. Complementary histological measures are routinely conducted notably to quantify hair cells loss with cochleogram. Ototoxicity studies can be performed in rodents (mice, rats), guinea pigs and large species. However, in undertaking, or at the very least attempting, all preclinical investigations within the same species, is crucial. This encompasses starting with pharmacokinetics and pharmacology efficacy studies and extending through to toxicity studies. In life read-outs include Auditory Brainstem Response (ABR) and Distortion Product OtoAcoustic Emissions (DPOAE) measurements that assess the activity and integrity of sensory cells and the auditory nerve, reflecting sensorineural hearing loss. Accurate, reproducible, and high throughput ABR measures are fundamental to the quality and success of these preclinical trials. As in humans, *in vivo* otoscopic evaluations are routinely carried out to observe the tympanic membrane and auditory canal. This is often done to detect signs of inflammation. The cochlea is a tonotopic structure. Hair cell responsiveness is position and frequency dependent, with hair cells located close to the cochlea apex transducing low frequencies and those at the base transducing high frequencies. The cochleogram aims to quantify hair cells all along the cochlea and consequently determine hair cell loss related to specific frequencies. This measure is then correlated with the ABR & DPOAE results. Ototoxicity assessments evaluate the impact of drug candidates on the auditory and vestibular systems, de-risk hearing loss and balance disorders, define a safe dose, and optimize therapeutic benefits. These types of studies can be initiated during early development of a therapeutic solution, with ABR and otoscopic evaluations. Depending on the mechanism of action of the compound, studies can include DPOAE and cochleogram. Later in the development, a GLP (Good Laboratory Practice) ototoxicity study may be required based on otic related route of administration, target, or known potential otic toxicity.

## 1 Introduction

An estimated 1·57 billion (95% uncertainty interval 1·51–1·64) people globally had hearing loss in 2019, accounting for one in five people (GBD 2019 Hearing Loss Collaborators, 2021). Hearing has been identified by the WHO as the third largest cause of years lived with disability globally and the most frequent sensory deficit in human populations (World Report on Hearing, 2021). In the 2021 World Report on Hearing, the WHO stated that Hearing is a key component of human intrinsic capacity; it is the sense most relied upon to communicate and engage with others. “Any decline in hearing capacity at any point during the life course, if not addressed in a timely manner, can adversely affect day-to-day functioning.”

A recent survey (HHTM, 2021) revealed that a third of American over 50 reported that hearing loss significantly affects their mental well-being. A majority (55%) report a discernible negative influence on their capacity to derive enjoyment from entertainment. Substantial percentages also highlight adverse effects on other domains, including 42% experiencing a detrimental impact on their social interactions, 40% on personal relationships, 32% on mental health, and 31% on the ability to carry out routine day-to-day activities.

Despite the recognized importance of hearing loss, the number of therapies addressing its reversal and prevention remains relatively small as compared to vision loss. Historically, the primary solution to address auditory deficits has been the use of medical devices such as hearing aids and cochlear implants.

These devices do improve the lives of patients. However, they are costly, inefficient in noisy environments, and often provide noisy unintelligible sounds (conversational hearing deficits). Further, they do not treat the cause.

In the last 10 years, there has been increased interest in pharmaceutical therapeutic targets in the auditory field (Cousins, 2022) allowing new entrants into this underserved clinical domain (Hammill and Campbell, 2018). Many forms of hearing loss are mediated by the death of hair cells and the subsequent loss of synapses connecting hair cells to auditory nerve fibers in the inner ear. The biological and molecular mechanisms involved in this sensory cell death are a topic of a great deal of recent research. Unfortunately, several failures of drug candidates in clinical phase have dampened the interest of pharmaceutical companies in the field. The main encouraging pathway is currently gene therapy, among those targeting genetic deafness caused by otoferlin mutation, with promising results in clinical trials (Amariutei et al., 2023; Ha and Avraham, 2023; Lv et al., 2024). Though otoferlin-related hearing loss is very rare, accounting for only 1–8% of cases of hereditary deafness (Ford et al., 2023), the results offer hope for treating other genetic forms of deafness. These encouraging results could spur pharmaceutical investment in this extremely underserved market.

Remarkably, only one drug has been approved and is marketed for a hearing disorder indication: in September 2022, PEDMARK® (sodium thiosulfate injection) was the first and only FDA (Food and Drug Administration) approved pharmacological treatment for hearing loss. It has also been approved in Europe as Pedmarqsi™, May 2023. Its indication to “Reduce the Risk of Ototoxicity Associated with Cisplatin in Pediatric Patients with Localized, Non-Metastatic Solid Tumors” is related to the thiosulfate ability to chelate cisplatin. Therefore, the activity of sodium thiosulfate is due to its ability to bind to cisplatin and prevent or reduce the ototoxic effects of cisplatin, without acting on auditory functions.

Interestingly, the first approved drug in a hearing disorder’s indication is a drug to prevent cisplatin ototoxicity. The landscape of hearing disorder therapeutics featured 23 compounds in clinical trials and a substantial preclinical pipeline with at least 56 assets. Among these, 7 in the clinical phase and 11 in earlier stage were targeting prevention or treatment of drug induced ototoxicity, and more specifically ototoxicity of 2 drugs: cisplatin and aminoglycosides (Isherwood et al., 2022; Table 1).

**Table 1:** Drug candidates in clinical development for drug induced hearing loss.

Drug candidate	Drug developer	Indication	Route of administration	Mechanism of action	Status
Sodium thisosulfate (STS) Pedmark^s^ in US Pedmarqsi™ in EU	Fennec Pharma	Cisplatin: Reduce the Risk of Ototoxicity Associated with Cisplatin in Pediatric Patients with Localized, Non-Metastatic Solid Tumors	IV	STS inactivates cisplatin through covalent binding	Approved and marketed
DB-020 (STS)	Decibel Therapeutics Regeneron	DIHL Cisplatin	TT	STS inactivates cisplatin through covalent binding	Phase 1b
SPI-3005	Sound Pharmaceuticals	DIHL aminoglycosides	oral	Mimic and inducer of gluthation peroxydase	Phase 2
ORC-13661A	Oricula Therapeutics	DIHL Aminoglycosides	oral		Phase 1
LPT99	Spiral Therapeutics	DIHL Cisplatin	TT	APAF-1 inhibitor	Phase 1
SENS-401	Sensorion	DIHL	Oral	5-HT3 receptor antagonist and calcineurin inhibitor	Phase 1
ACOU085	Acousia Therapeutics	DIHL cisplatin	TT	KCNQ4 Receptor agonist	Phase 2a

This focused effort on developing otoprotective drugs that can minimize the ototoxicity of already approved drugs highlights the importance of screening for ototoxicity during drug development. Though, assessment of effects on the eye are part of general toxicity studies and are described in the health authorities Guidelines assessment of effects on hearing functions remains neglected in drug development. Understandably, the inner ear is a complex organ. The inner ear is difficult to reach, embedded in the temporal bone, difficult to harvest and process. The techniques and methods require state-of-the-art technology and a broad and deep expertise in neurosciences and otology. Research laboratories serving this therapeutic area must be able to combine sophisticated surgical approaches for specific otic routes of administration, sampling of the inner ear fluids, electrophysiology, histology, and specific expertise in analysis and interpretation. However, considering the devastating nature of auditory dysfunction and hearing loss, such complexities should not deter companies from preclinically testing for ototoxicity as part of their safety assessment of novel drugs.

The aim of this narrative review is to raise the awareness of ototoxicity for researchers, drug and biotherapy developers, physicians, as well as health authorities and to formulate some recommendations based on available guidelines and own experience.

## 2 Ototoxicity

### 2.1 Definition- mechanism of action

Ototoxicity is defined as damage to the inner ear, targeting cochlear and vestibular structures and sensory function, due to exposure to certain pharmaceuticals, chemicals, and/or ionizing radiation (Steyger et al., 2018; Steyger, 2021) (the consensus of the Ototoxicity Working Group of Pharmaceutical Interventions for Hearing Loss, 2018).

The symptoms associated with ototoxicity are sensorineural hearing loss, tinnitus, aural fullness, dizziness, and vertigo (Altissimi et al., 2020). They can be temporary and reversible or permanent (Lanvers-Kaminsky et al., 2017; Watts, 2019). Symptoms can present gradually, simultaneously, in succession or individually (one at time). The onset can be immediate or delayed, even up to weeks from exposure to the trigger. The onset and severity are often dose-dependent and cumulative (World Report on Hearing, 2021). Many factors can exacerbate the risk of ototoxicity such as comorbidities, inflammation, kidney damage, and oxygen depletion, age, noise exposure or drug interaction (Coffin et al., 2021; World Report on Hearing, 2021).

Drug classes most associated with ototoxicity include antibiotics, such as aminoglycosides (gentamicin, kanamycin, …), and macrolides (azithromycin) (Rybak et al., 2021), platinum-based chemotherapeutic agents (cisplatin) (Karasawa and Steyger, 2015; Campbell and Le Prell, 2018; Hammill and Campbell, 2018; Barbieri et al., 2019; Reynard and Thai-Van, 2023), loop diuretics, such as furosemide (Ding et al., 2016; Joo et al., 2018; Altissimi et al., 2020; Skarzynska et al., 2020), antimalarial drugs such as chloroquine and hydroxychloroquine (Altissimi et al., 2020; Coffin et al., 2021; Jozefowicz-Korczynska et al., 2021), nonsteroidal anti-inflammatory drugs (NSAIDs) and acetylsalicylic acid (Ganesan et al., 2018; Altissimi et al., 2020). In addition, other drugs have been identified to be potentially ototoxic such as capsaicin, cimetidine, epinephrine, hydroxyzine, and sucralfate as possible candidate (Barbieri et al., 2019) and some immunosuppressant drugs, such as tacrolimus (Franz et al., 2022) and potentially cyclosporine (Waissbluth, 2020). During the COVID-19 pandemic, several drugs have been repurposed as therapeutics agents against COVID-19, not only hydroxychloroquine already cited but also anti-viral therapy (ritonavir, remdesivir), interferons, and anti-parasitic such as ivermectin (Coffin et al., 2021), all identified as potentially ototoxic.

Although ototoxicity and cochleotoxicity mechanisms of action are not fully elucidated, much progress has been made in identifying otoprotective solutions and/or drug replacement with reduced or no ototoxicity. The physiological isolation of the intricate hearing mechanism within the cochlea also poses transport barriers to ototoxic and otoprotective drug alike. Briefly, to be cochleotoxic, drugs have to enter the inner ear, and cross the blood labyrinth barrier (BLB), including the blood-perilymph and blood-strial barriers. Passage of drugs across the BLB depends on their physical and chemical properties (their lipophilicity, polarity, and size) and on the mechanism involved: active (transporters) and passive transport processes (Salt and Hirose, 2018). In addition, this passage could be enhanced by different factors (inflammation, infection, structural damage, and integrity of vessels). Once inside the cochlea, they can directly act on hair cell membrane receptors and/or enter hair cells using either the mechanoelectrical transducer (MET) channels located at the tips of the stereocilia or other ion channels (Kitcher et al., 2019; Coffin et al., 2021). Ototoxic agents can damage auditory hair cells, the spiral ganglion neurons and nerve fibers, and auditory neurons (Lin et al., 2021). Several excellent reviews extensively describe cochleotoxicity mechanism (Kitcher et al., 2019; Coffin et al., 2021).

### 2.2 Ototoxic drugs and epidemiology

The most studied ototoxic drugs include aminoglycosides (Jiang et al., 2017) and platinum-based antineoplasics drug classes (Lee et al., 2021), probably because of their wide use. The aminoglycosides are still among the most frequently used antibiotics worldwide (Xie et al., 2011), especially due to recurrent and resistant tuberculosis and their low price. In recent years, promising research has allowed a better understanding of the mechanism of action of their ototoxicity and will hopefully lead to the next generation of aminoglycosides, less ototoxic (Xie et al., 2011; Zhanel et al., 2012; Huth et al., 2015; Quirke et al., 2022).

Cisplatin is a major chemotherapeutic agent currently used against solid tumors. Cisplatin has a range of serious side effects, such as nephrotoxicity, neurotoxicity, and ototoxicity (Karasawa and Steyger, 2015). Cisplatin induces the death of sensory cells in the human cochlea, leading to permanent hearing loss because of the inability of these cells to regenerate. Cisplatin’s ototoxicity has been extensively described and published due to its irreversible character, seriously impacting patient quality of life. Despite these adverse effects, its efficacy against some cancers makes it difficult to replace especially in children, where the ototoxicity is even a more detrimental adverse event. Although some platinum-based alternatives with lower ototoxicity have been explored (Kros and Steyger, 2019; Mamillapalli et al., 2021), the unique efficacy of cisplatin complicates the prospect of substitution. As a dose-dependent relationship between a higher cumulative dose and a higher incidence of hearing loss has been established (Breglio et al., 2017), one option could be the reduction of cisplatin dose. Another option may be to change the frequency of administration, without compromising cisplatin efficacy. Alternative dosing with lower amounts per dose may reduce cisplatin accumulation in the cochlea and may potentially lead to less ototoxicity while retaining its antineoplastic properties. Several studies compared the standard of care regimen of high dose once every 3 weeks (100 mg/m^2^), versus low dose weekly cisplatin (30–40 mg/m^2^) (Szturz et al., 2017, 2019). Current evidence is insufficient to demonstrate a meaningful efficacy difference between the two dosing regimens and the three-weekly high-dose regimen and is therefore unlikely to alter the standard of concomitant chemotherapy (Szturz et al., 2017, 2019).

This sad reality has spurned a large investment in developing otoprotective therapies (Table 1).

The number of approved ototoxic drugs is not known, estimated as somewhere between 150 and 600 (Reynard and Thai-Van, 2023), with 200 as an average often published. In a 2020 review, Rizk et al. identified 194 systemically administered medications associated with ototoxicity (Rizk et al., 2020). The authors reported their difficulty in adequately querying the databases. For example, querying one of the databases used in this analysis for the term “ototoxicity” did not encompass amiodarone; however, a query for the term “bilateral vestibulopathy” yielded only amiodarone as a result.

Accordingly, it is difficult to assess the number of ototoxic drugs, as ototoxicity is typically reported after drug approval and then only as adverse event.

Pharmacovigilance ensures safe and effective use of medicines, the establishment of adverse drug reactions (ADRs) reporting systems, data bases, and risk assessment procedures. Legislation in the European Union, the United States, and most other countries requires regulatory authorities, sponsors and pharmaceutical companies to collect and store adverse drug reaction reports in a safety database. Mechanisms to collect adverse effects centrally are in place. In Europe the EudraVigilance system, a European database containing suspected adverse drug reactions, allows healthcare professionals and patients themselves to report side effects. In the US, the FDA has implemented the Adverse Events Reporting System (FAERS) and in Japan the Pharmaceuticals and Medical Devices Agency (PMDA) has implemented the Japanese Adverse Drug Event Report (JADER) database. The analysis of these data can lead to addition of the adverse effects in the labeling, and sometimes to market withdrawal. Guidance and standards for ADRs databases are issued by the International Conference on Harmonization (ICH E2B). Adverse events and medication errors are coded using terms in the Medical Dictionary for Regulatory Activities (MedDRA) External Link Disclaimer terminology. Side effects are classified in reaction groups. The ear reaction group is “ear and labyrinth disorders.” Reports are available to search for a medication’s or active substance’s suspected side effects (also known as adverse drug reactions). For each drug, searching for “ear and labyrinth disorders,” gives a list of adverse effects which raises some questions (Szczepek and Stankovic, 2021). In this list, ototoxicity is identified as a side effect. Does it mean that inner ear disorders, deafness, or sudden hearing loss are not ototoxicity? (Coffin et al., 2021). Hopefully, the results obtained in the US and Europe databases give very closed results, with some differences. For example, a search for cisplatin returns a list of 32 reported suspected reactions in the EU database and 40 in the US database (Table 2), 30 of which are similar (Rizk et al., 2020). Among these different types of deafness are identified: deafness, deafness bilateral, deafness neurosensory, deafness permanent, deafness transitory, deafness unilateral, conductive deafness, but also sudden hearing loss. When a patient or a physician reports an adverse effect, it is not certain that he/she can differentiate these different types of deafness. In the US database, a report gives the possibility to search by reaction item, but not by reaction group. Searching by reaction item, “ototoxicity” gives 1,648 hits, searching for deafness gives 27,289, Data as of December 31, 2023. Then, selecting for each reaction item, “Case by generic name” displays a histogram of the number of cases by generic name, sorted by decreasing number (Table 3).

**Table 2:** Reported Suspected Reaction, in ear and labyrinth disorders reaction group, for cisplatin Eudravigilance data base (Europe) and in the FAERS data base (US).

Reported suspected reaction	Europe – Eudravigilance	US- FAERS
Acute vestibular syndrome	🗸	🗸
Auditory disorder	🗸	🗸
Auricular swelling	🗸	🗸
Autophony	🗸	🗸
Cerumen impaction		🗸
Conductive deafness	🗸	🗸
Deafness	🗸	🗸
Deafness bilateral	🗸	🗸
Deafness neurosensory	🗸	🗸
Deafness permanent	🗸	🗸
Deafness transitory	🗸	🗸
Deafness unilateral	🗸	🗸
Ear disorder	🗸	
Ear Congestion		🗸
Ear Discomfort		🗸
Ear Disorder		🗸
Ear Hemorrhage		🗸
Ear Pain	🗸	🗸
Eustachian Tube Dysfunction	🗸	🗸
Eustachian Tube Stenosis		🗸
Hyperacusis	🗸	
Hypoacusis	🗸	🗸
Inner ear disorder	🗸	🗸
Mastoid Effusion		🗸
Meniere’s Disease		🗸
Middle Ear Disorder		🗸
Middle Ear Effusion	🗸	🗸
Middle Ear Inflammation		🗸
Mixed Deafness	🗸	🗸
Motion Sickness	🗸	🗸
Neonatal Deafness		🗸
Neurosensory Hypoacusis	🗸	🗸
Otorrhoea	🗸	🗸
Ototoxicity	🗸	🗸
Paraesthesia ear	🗸	
Presbyacusis	🗸	🗸
Sudden Hearing Loss	🗸	🗸
Tinnitus	🗸	🗸
Tympanic Membrane Disorder	🗸	🗸
Tympanic Membrane Perforation	🗸	🗸
Vertigo	🗸	🗸
Vertigo Positional	🗸	🗸
Vestibular Disorder	🗸	🗸
Total number of reactions	32	40

Database (USA).

**Table 3:** Top 10 drugs in number of cases of ototoxicity and deafness –the US database FAERS.

Generic name	Number of ototoxicity cases	Number of deafness cases
Adalimubab		1,162
Etanercept		674
Cisplatine	310	522
Apixaban		442
Lenalidomide		430
Methotrexate		419
Sacubitril/vasartan		419
Aspirin		341
Tofacitinib citrate		327
Carboplatin	224	324
Etoposide	153	
Cyclosporine	106	
Amikacin	106	
Vincristin	105	
Vincristine sulfate	98	
Gentamycine	91	
Vancomycin	82	
Cyclophosphamide	76	

There are also no accurate figures on the prevalence of ototoxicity. This is partly due to ambiguity in the number of ototoxic drugs, and partly due to the lack of proactive hearing surveillance in patients receiving ototoxic drugs (Garinis et al., 2021). Incidence of ototoxic hearing loss is estimated to be up to 33% with aminoglycosides and 6–7% with furosemide. Up to 50% of those treated with injectable medicines (e.g., Amikacin and Streptomycin) for drug-resistant tuberculosis (DR-TB), could develop permanent hearing loss (World Report on Hearing, 2021). Moreover, studies have reported a high incidence rate of cisplatin-induced ototoxicity (CIO), with 40–60% of patients having various degrees of hearing loss and 18% facing severe to profound hearing loss after cisplatin treatment. In children, the situation is even more serious. When cisplatin accumulates at or above 400 mg/m^2^, more than 70% of children experience severe hearing loss, presenting hindered speech and language development (Chattaraj et al., 2023; Wang et al., 2023; World Report on Hearing, 2021).

## 3 Rationale for monitoring ototoxicity during drug development

Ototoxicity assessment in non-clinical development or monitoring during clinical studies is usually not required by Health Authorities, except if a drug is administered by otic route or if the drug belongs to a family of drugs known to be ototoxic or at risk of ototoxicity.

By contrast, assessing risks to the eye is part of the drug development process. Ophthalmological examination is included in the repeated dose toxicity guidelines, S4 ICH guidelines, even if briefly described (Onodera et al., 2015). In life, morphological examinations are performed but the functional measures of visual acuity or perception are not measured. These results should be provided to the histopathologists for examinations of the eye (part of the list of organs to be sampled).

Ocular toxicology applies to drugs administered topically, intraocularly, or systemically (Onodera et al., 2015). When a drug is developed for an ocular indication, the potential ocular toxic effects have to be assessed, whether the drug is administered on /in the eye or by systemic route, which is very rarely used for ocular indication.

Coming back to any drug candidate administered by a systemic route for any indication, the ocular examination must be performed by a board-certified veterinary ophthalmologist, uniquely qualified person, who also assists the study director in the design of the tests and the analysis (Onodera et al., 2015). In addition, the eyes are in the core list of organs to be sampled and examined during general toxicology studies, as recommended by the Society of Toxicologic Pathology (STP) (Bregman et al., 2003), but ear and auditory tissues are not part of this list.

By applying the same principles, hearing function should be examined during drug development to look for Szczepek and Stankovic (2021) undesirable otic effects when the ear is the target organ, and (Coffin et al., 2021) undesirable hearing effects from an agent applied in a systemic manner.

This viewpoint is espoused by the guideline “Non-clinical Safety Evaluation of Reformulated Drug Products and Products Intended for Administration by an Alternate Route. Oct 2015 – FDA.” (Non-clinical Safety Evaluation of Reformulated Drug Products and Products Intended for Administration by an Alternate Route, 2015) This guideline defines the main readouts for assessing the auditory function in toxicology studies by the otic route: the auditory brainstem response (ABR) and the cytocochleogram. This is the only guideline for drug development in the world mentioning this route specifically. In Europe, non-clinical local tolerance testing is intended to support human exposure to a drug product (both active substance and excipients) at contact sites of the body following clinical use. Such local tolerance testing should aim to support initial testing in clinical trials (Guideline on Non-clinical Local Tolerance Testing of Medicinal Products, 2015).

Does it apply to drug administered by trans-tympanic route? We can guess it does, but again only ocular, transdermal, cutaneous, and transdermal routes of administration are described in this guideline. Based on the number of drugs in development administered by the otic route, specifications on the otic route should be described in these guidelines. However, in the case of a drug candidate administered by a systemic route, even if this drug is intended to treat or prevent hearing disorders, it is not mandatory to assess the potential toxicity on the ear. Of course, any study showing otoprotective benefit of a drug would inherently be assessing ototoxicity if hearing gets worse instead of better, but it will not allow a characterization of toxic effects with respect to the target organs, dose dependence, relationship to exposure, and, when appropriate, potential reversibility, according to the ICH guidelines M3R2.

## 4 Ototoxicity monitoring during non-clinical phase

Ototoxicity testing should start during the pre-clinical phase of drug development. Before administration of a drug candidate to humans, the health authorities must be convinced that the drug can be safely administered in defined conditions (route of administration, dose, frequency...). For a long time, the safety of a drug candidate was mainly assessed in animals. Recently, both the European parliament (sept 2021) and the FDA (December 2022) have approved texts to reduce the use of animals in research (FDA Modernization 5 Act 2.0, 2022; Plans and Actions to Accelerate a Transition to Innovation Without the Use of Animals in Research, Regulatory Testing and Education, 2021). The reduction of the use of animals in research is not new, the initial proposal dates back to 1959, the principles of 3R were included in the European regulation adopted in 1986 and integrated into ICH guidelines M3R2 in 2009. In the USA, the FDA refers now to non-clinical (instead of preclinical) to include “a test conducted *in vitro*, *in silico*, or *in chemico*, or a nonhuman *in vivo* test, that occurs before or during the clinical trial phase of the investigation of the safety and effectiveness of a drug. Such tests may include cell-based assays, organ chips and microphysiological systems, computer modeling, other non-human or human biology-based test methods, such as bioprinting and animal tests.”

Although the laws are changing to reduce animal numbers, regulatory guidances are much slower to change. There are ongoing efforts to get new tests and techniques accepted by regulatory authorities. These new tests and techniques must be reproducible and translate to human findings. The FDA has implemented a qualification program to support the researcher in the qualification of their new tests. Thus, replacing the *in vivo* studies is not a reality today, but using alternative methods can at least predict early in the development the ototoxicity risk, in a rapid manner at lower costs and reduce the number of animals used for *in vivo* tests.

### 4.1 Non *in vivo* models

#### 4.1.1 In silico

*In silico* toxicology (IST) methods are computational approaches that analyze, simulate, visualize, or predict the toxicity of chemicals, usually based upon their chemical structure. The major *in silico* prediction methodologies include:

- QSAR (Quantitative Structure–Activity Relationship) approach: predicting toxicity with machine learning/statistical algorithms.
- Expert Rule-Based: predicting toxicity with empirical observations, literature, alerts, etc.
- Read-Across approach: predicting the toxicity based on the known toxicity of similar related substances.

Each methodology has its strengths and weaknesses, which often depend on the type of toxicological effect or mechanism being predicted (Myatt et al., 2018). *In silico* methods require no compound, reduce animal use, and can eliminate compounds earlier in the discovery process reducing costs and time to lead selection. This method is useful for screening multiple compounds, or to detect potential risks earlier. *In-silico* toxicology is already used in different domains of toxicology such as hepatotoxicity, cardiotoxicity, phototoxicity (guidelines ICHS10), mutagenicity (in the ICH M7 guideline, QSAR predictions for the Ames mutagenicity of drug impurities can be used for regulatory purposes).

Presently, there is a scarcity of IST methods to detect ototoxicity; our review has identified only 3 publications on this topic (Zhou et al., 2014; Zhang et al., 2020; Coffin et al., 2021), based on QSAR.

The standard methodology of this approach consists of 4 phases:

- Data collection: get a set of experimental data that includes both the chemical structure of the compounds and an ototoxicity label.
- Descriptor selection: choose relevant molecular descriptors (physico-chemical properties, topological properties…) that represent in a meaningful way the chemical structure with the aim of predicting its ototoxicity. This selection can be determined both by statistical algorithms and expert approaches.
- Model training: train a machine learning/statistical model (Support Vector Machines SVM, Neural Network, Decision Tree, Naïve Bayesian, Recursive Partitioning…) to predict ototoxicity based on selected descriptors. This model is trained on a subset of the collected data, the training set.
- Model validation: evaluate the model performances on the rest of the data, the test set. The aim is to verify the model’s ability to generalize its predictions to new compounds.

In 2014, Zhou et al. collected 919 molecules on HSDB and DrugBank, 572 of which were classified as ototoxic. Using statistical preprocessing methods (standard deviation, incompleteness, correlation, Genetic Algorithm, Monte Carlo method…), they selected 32 relevant descriptors on 237 available. By training several models and comparing their performances, they concluded that the one with the best accuracy score was a GA-CG-SVM with 85.33 and 83.05% for two independent test sets (Zhou et al., 2014).

In 2020, Zhang et al. collected 2,612 diverse chemicals, 897 of which were classified as ototoxic. Using statistical preprocessing methods (standard deviation, incompleteness, correlation, Genetic Algorithm…), they have selected 7 relevant descriptors on 140 available. By training several models and comparing their performances, they concluded that the one with the best accuracy score was a Naïve Bayes classifier with 90.2 and 88.7% for two independent test sets. To give concrete results, vidarabine is classified as ototoxic and has been well predicted, whereas glafenine is safe but has been predicted as ototoxic (Zhang et al., 2020).

In 2021, Coffin and Steyger compiled a list of drugs appearing on PubMed, and selected randomly in the test set 92 drugs, 21 of which were classified as ototoxic. They extracted from these substances their isomorphic SMILES (Simplified Molecular-Input Line-Entry), used as the input of their model. It describes the chemical structure as a series of fingerprints. They trained a Tanimoto model and obtained 75% of accuracy on their test set. Then, they tried to apply this model to 10,000 drugs from PubChem and predicted 180 ototoxins. Some of them were known in the literature (such as kanamycin), some others were real discoveries (vindesine, isradipine), but some substances were revealed to be false positives (dihydromyricetin) after testing in zebrafish (Coffin et al., 2021).

These encouraging results pave the way for other research and, hopefully, future ototoxicity modeling efforts will benefit from new machine learning approaches.

The development of IST protocols is a challenging task, leading to the creation of an international consortium of regulators, government agencies, industry representatives, academics, model developers, and consultants from various sectors to support the overall process. Guidelines from this consortium could also prove beneficial for initiatives related to ototoxicity (Myatt et al., 2018).

#### 4.1.2 Cell-based assays screening

The most widely used cell line in ototoxicity screening is the HEI-OC1 (House Ear Institute-Organ of Corti 1). These cells have been cloned, established, and characterized by the Kalinec group in UCLA (Kalinec et al., 2003). HEI-OC1 cells derive from the auditory organ of the transgenic mouse Immortomouse™. They express specific markers for cochlear sensory cells such as prestin, myosin 7a, Atoh1, BDNF, calbindin and calmodulin, but also markers of supporting cells like connexin 26 and fibroblast growth factor receptor (FGF-R) (Kalinec et al., 2016). Therefore, they are considered as precursors for cochlear cells. It has been shown that their response to ototoxic drugs is specific as compared to other, non-auditory cell lines such as HEK-293 or HeLa cells (Kalinec et al., 2016). Relatedly, the cell lines UB/OC-1/2 were equally generated from the Immortomouse™ and developed by Matthew C Holley, to provide screening tools for ototoxic reagents (Rivolta et al., 1998).

Some typical assays include cell proliferation, viability, cell death, apoptosis, and ROS production. The investigational compounds can be compared to a known ototoxic drug such as cisplatin, which inhibits cell viability and proliferation, while inducing apoptosis and ROS production.

More than 250 studies have been published in the last decade, using the HEI-OC1 cell line to screen for ototoxicity or otoprotection (Kalinec et al., 2016).

Although otic cell lines offer a restricted perspective because of their early developmental stage and different environment conditions compared to *in vivo* hair cells, they provide the advantage of faster assay speed and simpler experimental setup for screening ototoxic drugs.

The advantage of using the otic cell lines to screen for ototoxic drugs is the speed of the assay, ease of experiment setup, and cost. Screening of 20 compounds or less takes around 2 months, screening of 200 compounds, around 4 months (Cilcare’s data).

**Figure 1:**
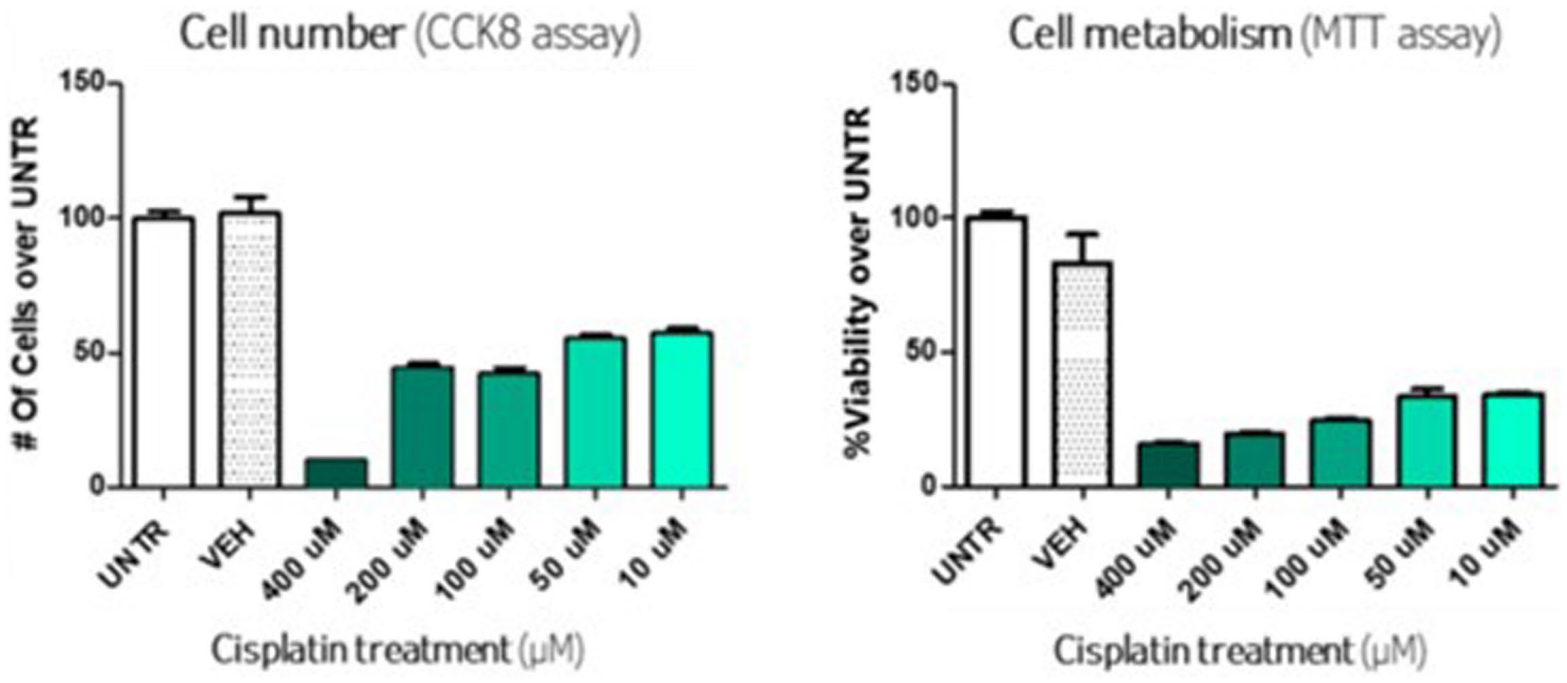
*In vitro* assay with cisplatin on otic cells line.

Many different conditions can be tested in parallel, such as different drug concentrations and read-outs. Among the test, the CCK8 assay, to quantify the viable cells and the MTT, to assess cell metabolism (measure of mitochondrial activity, which correlates with cell viability), are illustrated in Figure 1. In this model, cisplatin induced significant cell death, with a dose-effect ranging from 10 μM to 400 μM.

#### 4.1.3 Organoids

The term “organoid” means “organ-like.” Organoids are 3D structures derived from stem cells or cells progenitors which, on a much smaller scale, recreate important aspects of the 3D anatomy and multicellular repertoire of their physiological counterparts, and can summarize basic tissue functions.

Organoids from many organs have been developed, including pancreas, kidney, liver, thyroid gland, retina, ovary, and brain, offering an *in vitro* framework for studying drug pharmacology in a more biologically and pharmacokinetically relevant environment compared to cell culture.

In 2019, authors have shown that inner ear organoids offer the possibility of studying sensory cell types of human origin *in vitro* (Roccio, 2021). Recently, some researchers used an established protocol to generate cochlear organoids from murine Lgr5+ progenitor cells. Transcriptional signatures of maturing hair cells were apparent after 10 days of organoid differentiation, and during the course of differentiation, cells mimicked nearly all subtypes of supporting cells and hair cells in the newborn cochlea (Kalra et al., 2023).

Human organoid models will provide insight into unique human aspects of inner ear development and pathologies, which are impossible to gain from animal studies. The human induced pluripotent stem cell (hiPSC) technology and 3D cell culture aims to generate cochlear organoids containing neurosensory cells from the human inner ear. Otic neurosensory cells derived from cochlear organoids allow molecular screening to accelerate the discovery phase of molecules that may have potential in the protection of hair cells and/or auditory neurons. The development of this screening tool aims to limit the use of animal models and to closely emulate the human ear.

A benefit of organoids is their ability to mimic the organ or *vivo* tissue from which the cells that compose it are derived. This allows cell–cell and cell-matrix interactions. Nevertheless, a primary constraint in utilizing the organoid model for tissue generation is that, upon reaching a specific size, organoids cease to proliferate and develop a necrotic nucleus. To maintain the complexity of the organoids, it is necessary to prevent the internal appearance of the necrotic core leading to premature differentiation in the outer layers of the organoids. This phenomenon has been largely attributed to a lack of vascularization of organoids. It is therefore important to focus on the development of vascularized organoids (van der Valk et al., 2021).

Inner ear organoids, by its 3D structure and organization, constitute a highly relevant tool to evaluate potential ototoxic effects of drugs, after establishing reference data with proven ototoxic agent like an antibiotic of the aminoglycoside class (e.g., gentamycin) or a carboplatin molecule (e.g., cisplatin). In addition, as organoids also presented neural cells, it can be used as a model with auditory nerve fiber and cells damage induced by ouabain. In such models, the protective effects of investigational drugs can be evaluated.

Inner ear organoids exhibit a significantly higher level of complexity compared to otic cell cultures. They still lack some characteristics of complete human cochlea, and, unlike an animal model, organoids are not connected to an entire body system.

#### 4.1.4 *Ex-vivo* studies – the explants

Cochlear explants in neonatal rodents are an organotypic culture of the immature cochlea, facilitating the presentation of organized cellular structures within the inner ear, which are otherwise hard-to-access. Since the method was established by Sobkowicz et al. (1975), more than 500 hundred publications (search “cochlea and explant” in Pubmed) reported the use of this invaluable tool for studying and understanding cochlear physiology and function, including the evaluation ototoxic of drugs, as well as otoprotective treatments. During drug development, explants can be considered for a rapid and direct evaluation of potential ototoxic effects, enabling an efficient screening and selection between drug candidates. The cochlear explant studies have some great advantages but also some limitations.

One notable advantage of using explants is the preservation of the three-dimensional structure of the cochlea. Explants enable a rapid and simultaneous study of the drug candidate’s effect on cochlear hair cells, supporting cells, and neurons. It is possible to test a drug candidate in different protocols and at various concentrations. It is an ideal model to find an optimal dose for *in vivo* studies. Indeed, the lack of BLB can also be considered as a strength of this technique, overcoming the challenge of drug delivery into the inner ear (Nyberg et al., 2019). Overall, it accelerates screening and drug candidate selection for *in vivo* testing. Cochlear explants are thus an important step between *in vitro* and *in vivo* testing.

The limitations mainly include the preparation of explants which presents an important technical challenge, the dissections requiring patience and practice (Ogier et al., 2019). Secondly, readouts are limited to the cellular level and hearing cannot be assessed. Thirdly, the quantity of drug reaching the cochlear epithelium is not regulated by the presence of the BLB and the physiological state of the inner ear, as it would be *in vivo*. All the membrane and vascular barriers forming the BLB, limiting the drug diffusion from one fluid to another (Blood/perilymph, blood/endolymph, and endolymph/intrastrial fluid) (Nyberg et al., 2019) are absent in an organotypic culture of the organ of Corti, exacerbating the drug effects. Finally, the explant model does not reproduce the constant entry and clearance rate of drug after administration, at least initially (Plontke and Salt, 2018).

Cochlear explants are also used as an ototoxicity screen. Compounds showing toxicity in cochlear explants is indicative of the compound’s toxicity in adult cochlea. Explant toxicity triggers a recommendation for *in vivo* ototoxicity testing or removal as a drug candidate.

### 4.2 *In vivo* models

#### 4.2.1 Zebrafish

The zebrafish lateral line is a relevant model for understanding hair cell function and dysfunction (Harris et al., 2003; Ou et al., 2007; Olt et al., 2014). The lateral line is a system of mechanosensory hair cells on the body surface that allows fish to detect fluid displacement. The system consists of neuromasts, clusters of hair cells surrounded by supporting cells. These hair cells depolarize, as their stereocilia are displaced, and send signals to the brain with associated nerve fibers, similar to the functions of hair cells in the auditory and vestibular labyrinth. Lateral line hair cells are easily accessible for experimental manipulation and provide a rapid system for screening compounds for ototoxicity (Chiu et al., 2008; Owens et al., 2008; Hirose et al., 2011). There are three main limitations of the zebrafish lateral line model (Szczepek and Stankovic, 2021) Hair cells are able to spontaneously regenerate (Lush and Piotrowski, 2014), unlike mammalian hair cells. This limits the analysis window for protective drug effects. Protective effects would need to be evaluated prior to hair cell regenerative process start (Coffin et al., 2021) Immature and mature HCs coexist with different biophysical properties and probably with different protein components (Olt et al., 2014; Rizk et al., 2020) Similar to the explant model, the zebrafish lateral line model does not reflect the behavior of a drug in a mammal organism, as the drug is directly delivered in the water bath of the zebrafish and does not encounter the challenge of BLB crossing or constant elimination dur to inner ear fluid removal *in vivo*.

Although zebrafish use in hearing field is mainly related to lateral line study, Zebrafish have an inner ear that shares the same basic developmental mechanisms and auditory and vestibular functions as mammalian ears (Schuck and Smith, 2009). Inner ear hair cells are also able to regenerate after damage, similarly to those of lateral line. In the young larva, five of the six definitive sensory epithelia are already present in this species, including the one that performs the auditory function more specifically, the posterior macula (Bauer et al., 2021). The advantage of working at this stage is that the posterior macula contains a much smaller number of hair cells than in adults, which makes it easier to quantify them. Finally, the architecture of the larval ear is still simple, facilitates cell visualization in the entire embryo. This model can then be used to create a test process for screening many molecules *in vivo* in a relatively short time using individuals from the same clutch. As evaluation of zebrafish inner ear is more complex than the evaluation of its lateral line, it has been less used.

#### 4.2.2 Rodents and large species

Whether it is to assess efficacy or safety of a drug candidate, the contribution of animals, especially mammals, remains essential for the translation of results obtained at the bench to human. Despite the progress made *in vitro, ex vivo,* and the promising modeling of data, assessment of ototoxicity in animals remains the only reliable test to verify the non-ototoxicity of drug candidates. The primary challenge lies in selecting a validated, reproducible, and accurate model to obtain predictive data (Denayeretal, 2014; McGonigle and Ruggeri, 2014; Barré-Sinoussi and Montagutelli, 2015; Robinson et al., 2019).

There is no standard design for ototoxicity studies. Hundreds of publications describe ototoxicity models to assess cisplatin or aminoglycosides ototoxicity, yet there is still no consensus (Lin et al., 2021). This underscores the complexity of establishing a universal single model. However, identifying key parameters holds promise for developing a more predictive model with results that are translatable.

Hearing sciences require an acoustic laboratory, dedicated rooms, or cabinets, with a controlled noise level and specific equipment for the auditory measures like ABR and DPOAE (Distortion Product OtoAcoustic Emissions). Other specialized procedures include otoscopy, otic drug delivery, and otic fluid sampling. Personnel with advanced skills in these techniques and knowledge in electrophysiology, electroacoustics and neurosciences are essential. As an example, the local administration of a drug directly into the middle or inner ear via trans-tympanic administration, round window injection, posterior semicircular canal injection or intracochlear surgery should be conducted by experts who routinely perform these techniques in different species. This reduces failure rate and data misinterpretations.

ABR and DPOAE equipment can be custom made but is commercially available. In both cases, the installation of the equipment must be validated, and the equipment should be checked and calibrated at least before each study.

Most companies work with CROs specialized in these domains, as they do for ocular toxicity (Short, 2021). There are few CROs in the world offering ototoxicity services, and fewer with GLP capabilities as well. Those specializing in otic studies must have their own historical control data for the species they work with. As detailed later in this review, there are no standard data, as there are for heart or blood toxicity. It is imperative when working with an otic specialized CRO, that the outline of the study is as detailed as possible, and the study director understands what is needed by the client. Given the absence of specific regulatory guidance for otic studies, a transparent dialogue between the client and CRO is essential to tailor a study meeting regulatory needs.

The key points to consider when designing ototoxicity studies are (Szczepek and Stankovic, 2021) the objective of the study (Coffin et al., 2021) the animals -species, number, sex- (Rizk et al., 2020) the dosage regimen –route, frequency, dosage, duration, if the route is trans-tympanic or intraochlear, in one ear or both ears (Lanvers-Kaminsky et al., 2017) Readouts – which ones, frequency of measure.

##### 4.2.2.1 Species selection

During drug development, it is preferrable, but not always feasible, to conduct all preclinical work in the same species, from PK/PD studies to toxicology studies. This facilitates analyses and interpretation of results across studies. Each species has its own advantages and disadvantages. Some species are preferable in specific toxicological models, e.g., the rabbit for eye or dermal toxicity. This is the case for cats in the vestibule domain or cochlear implant models. These models have a historical database. Therefore, changing to another species takes time, creating a historic bias.

Rodents are very commonly used in hearing studies; mice, rats, Guinea pigs, chinchillas, and gerbils (Lin et al., 2021). Mice are the most used species in genetic and inner ear research. The Guinea pig has been a preferred model to test new therapies (Naert et al., 2019) and for pharmacokinetics studies due to the ease of delivering drugs into the inner ear, and the larger volume of inner ear fluids compared to mice and rats.

Large species can also be used such as dog, cat or preferably non companion animal such as swine and sheep (Wang et al., 2011). Furthermore, non-human primates (NHP) are also used, especially for gene therapy.

Rodent cochlea operates according to standard mammalian principles. Nevertheless, there are some differences that can affect species selection. Even if the hearing abilities of humans and laboratory animals overlap, hearing sensitivity is species dependent, especially in the high- and low-frequency ranges (Heffner and Heffner, 2007; Lin et al., 2021; Domarecka and Szczepek, 2023). The optimal hearing frequency of mice, rats, and guinea pigs is higher than the optimal hearing frequency of humans. Similarities and differences in the auditory anatomy of rodents and humans, such as cochlear turns, sensory hair cells, and central auditory system (Lin et al., 2021) are presented in Table 4. For cochlea turns, human, mice and rats have two and a half turns, while swine and guinea pigs have three and a quarter turns.

**Table 4:** Comparison of elements of the auditory anatomy of mice, rats, guinea pig and human.

Species	Tympanic membrane surface area (mm^2^)	Tympanic membrane thickness (mm)	RWM thickness, (μm)	Number of turns	Number of IHCs	Number of OHCs	TT injection volume	Endolymph volume (μL)	Frequency range (hearing)	References
Human	50–88	0,6	~70	2,5	3,200–3,400	10,000–12,000	400–600	30–34	0,02 –20 kHz	El Kechai et al. (2015), Glueckert et al. (2018), Salt and Hirose (2018), Ma et al. (2019)
Guinea pig	23,9	0,035	40	3,25	~1900	~7,000	50–70	1,2–1,5	0,1–54	Thorne et al. (1999), El Kechai et al. (2015), Salt and Hirose (2018), Trinh et al. (2022)
Rat	11	0,0135	10–12	2,5	977**	~3700**	30	0,39	0,1–64 (56–72)	Burda et al. (1988), Thorne et al. (1999), Wang et al. (2016), Nordang et al. (2003)
Mouse	3,9	~0,0135	<10	2,5	700 757**	2,400 2562**	5	0,78 μL	~5–80	Burda et al, 1988, Thorne et al. (1999), Bohne and Harding (2011), Hirose et al. (2014), Wang et al. (2016), Glueckert et al. (2018)

**Numbers for Wistar rat and NMRI mouse. Burda et al. (1988).

The size of the ear is obviously different and consequently the volume of inner ear fluids or drug that can be administered is also very different. Chinchillas were frequently chosen due to the size of their bulla; but their utilization in toxicology studies is not common. For toxicology studies, Guinea pigs, rats and mice are more likely choice, both for trans-tympanic and other otic administration routes.

Sensitivity is both species and drugs dependent. Guinea pigs are very sensitive to inflammation. Compared to mice and rats, Guinea pigs inner ears are more sensitive to toxic drugs (Yorgason et al., 2011). Guinea pigs and chinchillas show higher susceptibility to aminoglycosides, than adult rats and mice (Lin et al., 2021). Although adult mice and rats are resistant to some ototoxic drugs, they are still widely used in studies of drug-induced hearing loss. For example, Guinea pigs were shown to be more sensitive to kanamycin and cisplatin than mice (Poirrier et al., 2010).

In addition, sensitivity can vary between stains for a same species. CBA/Ca mice show stable hearing thresholds in advanced age (12–18 months), therefore they are suitable for experiments involving chronic exposure to ototoxic agents and constitute the reference mouse strain in the hearing field (Bramhall, 2021). One of the most used mouse strains is the C57BL/6, as it is the main genetic background of transgenic mice. However, C57BL/6 mice are known to be deaf early in life, due to a mutation of cadherin 23 (Kane et al., 2012), so the strain should be avoided in chronic ototoxicity studies.

There are many factors which should be considered in the species and strain selection (Lin et al., 2021), but generally, the preferred species/strains to assess ototoxicity are Wistar rats- for the historical database in toxicology, CBA/CaJ mice, and Hartley Guinea pigs. Notably, both pigmented and albino guinea pig (Hartley Guinea pigs) strains are used in auditory research. The effect of pigmentation, including a protective role of melanin, has been described in noise- and drug-induced hearing loss (DIHL) in other rodent species (Wu et al., 2001; Murillo-Cuesta et al., 2010; Naert et al., 2019).

The number of animals must be sufficient to ensure statistical power. For regulated GLP studies, the number of recommended animals is 10 per sex per group. In non GLP studies, 10 animals per group is also recommended but one sex may be selected in early studies, except if the drug is known to have gender-specific effect.

##### 4.2.2.2 Dosage regimen

Significant advances in harmonization of nonclinical safety studies for the conduct of human clinical trials for pharmaceuticals have already been achieved thanks to the ICH (The International Council for Harmonization of Technical Requirements for Pharmaceuticals for Human Use). However, differences remain between the 3 regions, USA, EU, and Japan, such as in the duration of toxicology studies. Before initiating a GLP ototoxicity study, it is recommended to validate the design with the health authorities.

To assess the ototoxicity of drugs during their development, the study should be conducted in clinically relevant conditions. The route of administration must reflect or be the same as the intended clinical route, and the formulation with the excipients should be the same, for the GLP studies, or very close otherwise (European Guideline, 2010).

As it is well known that ototoxicity is influenced by the dose, various doses must be tested. In a GLP toxicity study, 3 doses are usually tested: a low dose, usually corresponding to the pharmacological active dose, a high dose, and an intermediate dose, to assess the dose effect (ICH S3A-ICH HARMONISED TRIPARTITE GUIDELINE a Guidance for Assessing Systemic Exposure in Toxicology Studies, 1994; European Guideline, 2010). The higher doses are also defined by drug properties, notably the maximal solubility. For non GLP studies, 2 doses are recommended: the highest possible (i.e., maximal solubility) dose and the pharmacological dose. A reversibility period is needed, to check if the potential effects are transitory or permanent. In addition, hearing assessment must be conducted with a sufficient delay from drug administration, as some ototoxic drugs, like aminoglycosides, are known to induce late ototoxic effects, within weeks after a single treatment.

##### 4.2.2.3 Ototoxicity biomarkers in mammals

According to FDA-NIH Biomarker Working Group, a biomarker is “a defined characteristic that is measured as an indicator of normal biological processes, pathogenic processes or responses to an exposure or intervention.” (FDA-NIH Biomarker Working Group, 2016). In recent years, research has intensified to highlight molecular markers for both the diagnosis and prognosis of hearing disorders and their therapy, and few have been identified (Bellairs et al., 2022). Some scientific studies have tried to define blood biomarkers of hearing loss to have a translational measurement tool both for the diagnosis of the pathology and the prognosis of therapies. Prestin, a protein expressed in the outer hair cells, is used as a marker of outer hair cell integrity. Currently, many studies are underway searching for translational biomarkers in hearing pathologies (Rüttiger et al., 2017).

###### 4.2.2.3.1 Clinical signs and otoscopic examinations

Standard ear examination techniques for tolerability/dose range and pivotal ototoxicity studies include otoscopic examination. Otoscopy allows one to observe the tympanic membrane and auditory canal enabling the integrity and appearance of the tympanic membrane to be assessed. The scoring criteria often includes tympanic membrane bulging, opaque appearance, vascular dilation and perforation. Other otoscopic findings such as signs of irritation in the ear canal or presence of ear wax will be mentioned as additional comments. Additionally, clinical signs are regularly observed over the course of an ototoxicity study. Observations such has head tilt or nystagmus can be signs related to ototoxicity.

###### 4.2.2.3.2 Electrophysiology -electroacoustic

During electroacoustic measurements, animals are anesthetized and placed in an acoustic chamber to completely isolate them from exterior noise.

Auditory functions can be assessed by two non-invasive measures: ABRs and DPOAEs, the combination of which allows a differential diagnosis of the sites of dysfunction. ABR assessments are often performed during GLP ototoxicity studies and are part of the FDA Guidelines. This practice is understandable, as ABR is a valid and reliable preclinical assessment of auditory function (Abernathy et al., 2015) and is among the few measures that can be effectively translated to human.

**Figure 2:**
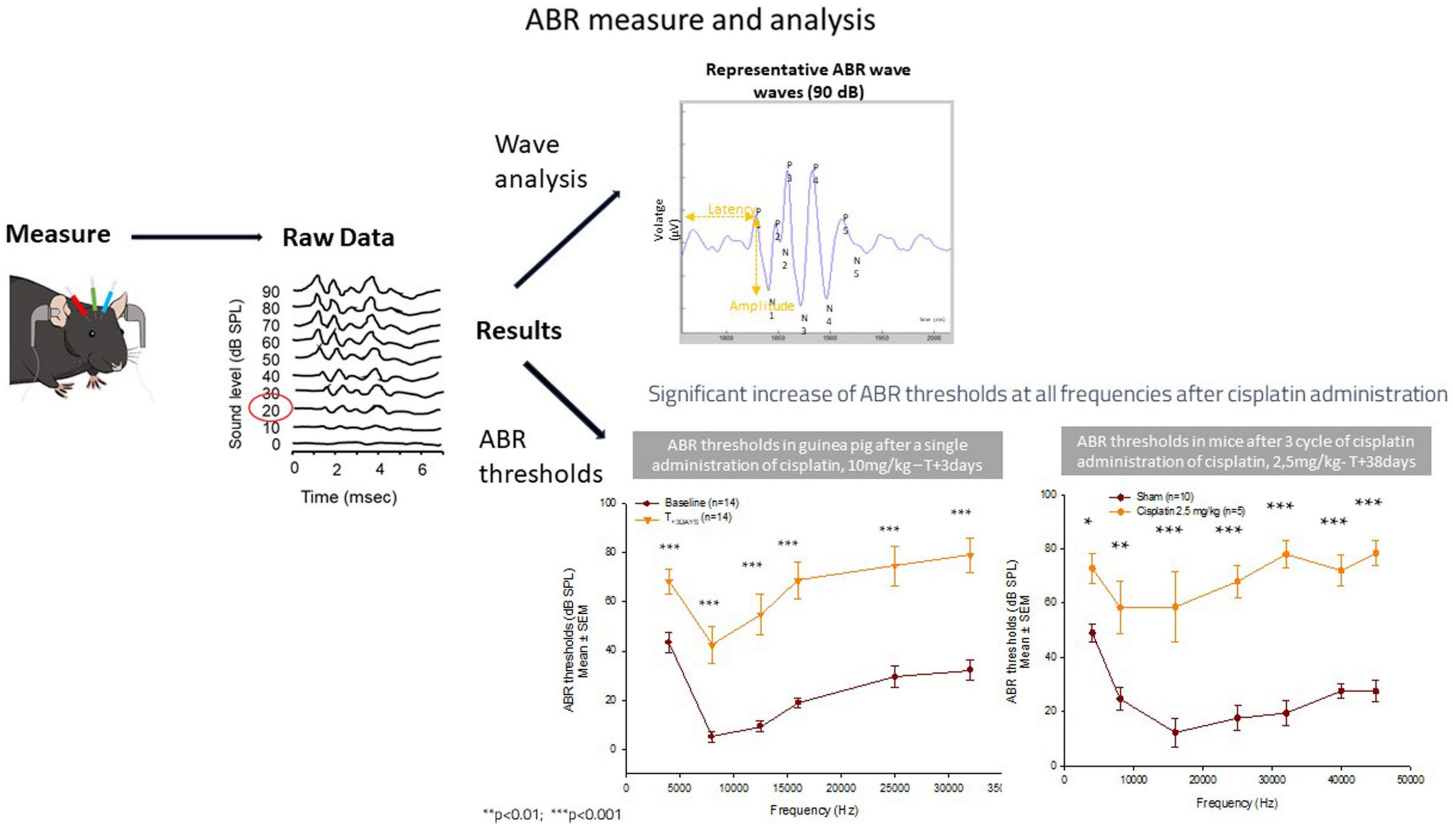
ABR measure and analysis.

ABRs are used in hearing programs and measures the functionality of the cochlea and neural pathways to the brain. Electric potentials obtained after auditory stimulus are recorded from three electrodes placed subcutaneously in the animal: vertex of skull, mastoid bone, and ground electrode. ABRs reflect the synchronous discharge of multiple neurons after the stimulus. ABRs consist of positive peaks, called waves, from 1 to 5 in rodents. The waves are characterized by their amplitudes and latencies. The ABR measure leads to the determination of the ABR threshold and ABR threshold shifts, and the analysis of the ABR waves (Figure 2). The first parameter analyzed is the ABR threshold (expressed in decibel): when the level of the stimulus is decreased, the amplitude of the response gets smaller, and the latency increases, the lowest intensity at which the response is present is considered the hearing threshold, which informs on the hearing sensitivity per frequency. An ABR threshold increase corresponds to a hearing loss. An elevation of the threshold is a typical effect of an ototoxic drug. The ABR thresholds shifts allow to compare the threshold between two groups or between the baseline measure and the measure obtained after a drug administration, a noise administration or simply over time. The first ABR wave – Wave 1- represents the summed activity of the auditory nerve fibers contacting the inner hair cells (IHCs). The amplitude of the Wave 1 is used to identify cochlear synaptopathy. Cochlear synaptopathy, the loss of synaptic connections between IHCs and auditory nerve fibers, has been documented in animal models of aging, noise, and ototoxic drug exposure, three common causes of acquired sensorineural hearing loss in humans. In animals with normal auditory thresholds, the amplitude of ABR Wave 1 recorded to supra-threshold stimulus levels appears to be a sensitive indicator of synaptopathy and is highly correlated with synapse counts (Furman et al., 2013; Kujawa and Liberman, 2015).

Currently synaptopathy can only be confirmed in humans through post-mortem temporal bone analysis. To assess cochlear synaptopathy in living humans (Bramhall, 2021), a combination of different clinical measures is currently being investigated. When middle ear conduction, OHC function, and threshold sensitivity are all normal, ABR Wave I measures are used to predict synaptopathy (Bramhall et al., 2019).

Many parameters can influence the ABR measure. Therefore, the design and protocols for conducting this measure must be standardized to ensure reproducibility and accuracy of the results. A recent article gives recommendations on planning and performing the ABR with a focus on mice and rats (Domarecka and Szczepek, 2023), to which scientists can refer to better understand how to measure ABR in rat and mice.

There is no standard or universal reference ABR threshold curve for each species, but the obtained curve has a U shape, the bottom of the U denotes the species hearing optimum. For this reason, the lab must have its own historical control data, with small standard deviations, and always perform baseline measures before any intervention on the animals, to make sure each animal has good hearing before treatment.

DPOAE is a measure of OHC integrity. DPOAEs are acoustic signals created and amplified by the cochlear epithelium and measured in the ear canal. DPOAEs depend on the biological motors in OHCs, which amplify sound-evoked cochlear vibrations. They do not depend on IHCs or auditory nerve fibers. DPOAEs are not explicitly included in the FDA Guidelines; however, their incorporation into an ototoxicity study enables a comprehensive assessment of the potential impact of the tested compound on outer hair cells.

###### 4.2.2.3.3 Histology

Histology is a critical endpoint for the evaluation of drugs in general toxicology studies, this is also true for ototoxicity studies. As a reminder, the FDA recommends that “*for drugs intended to reach the inner or middle ear, toxicity studies should also include the evaluation of the auditory brainstem response as well as the evaluation of microscopy of relevant otic tissues including a cytocochleogram.”*

There are challenges to inner ear histology: (Szczepek and Stankovic, 2021) harvesting, because the inner ear structures are buried in the hardest bone of the human body (Coffin et al., 2021) decalcification of the protective bone encasement of the tissue (Rizk et al., 2020) surface preparation of the organ of Corti.

####### 4.2.2.3.3.1 Cochleogram

The cochleogram allows histopathological assessment of ototoxic damage on both inner and outer hair cells of the cochlea as a function of their tonotopic location. The cochlea is composed of 4 rows of hair cells: 1 row of inner hair cells directly connected to the auditory neurons (activity is measured with ABRs) and 3 rows of outer hair cells (activity measured with the DPOAEs); depending on their position in the cochlea either close to the apex or to the base, they transduce low or high frequencies, respectively.

**Figure 3:**
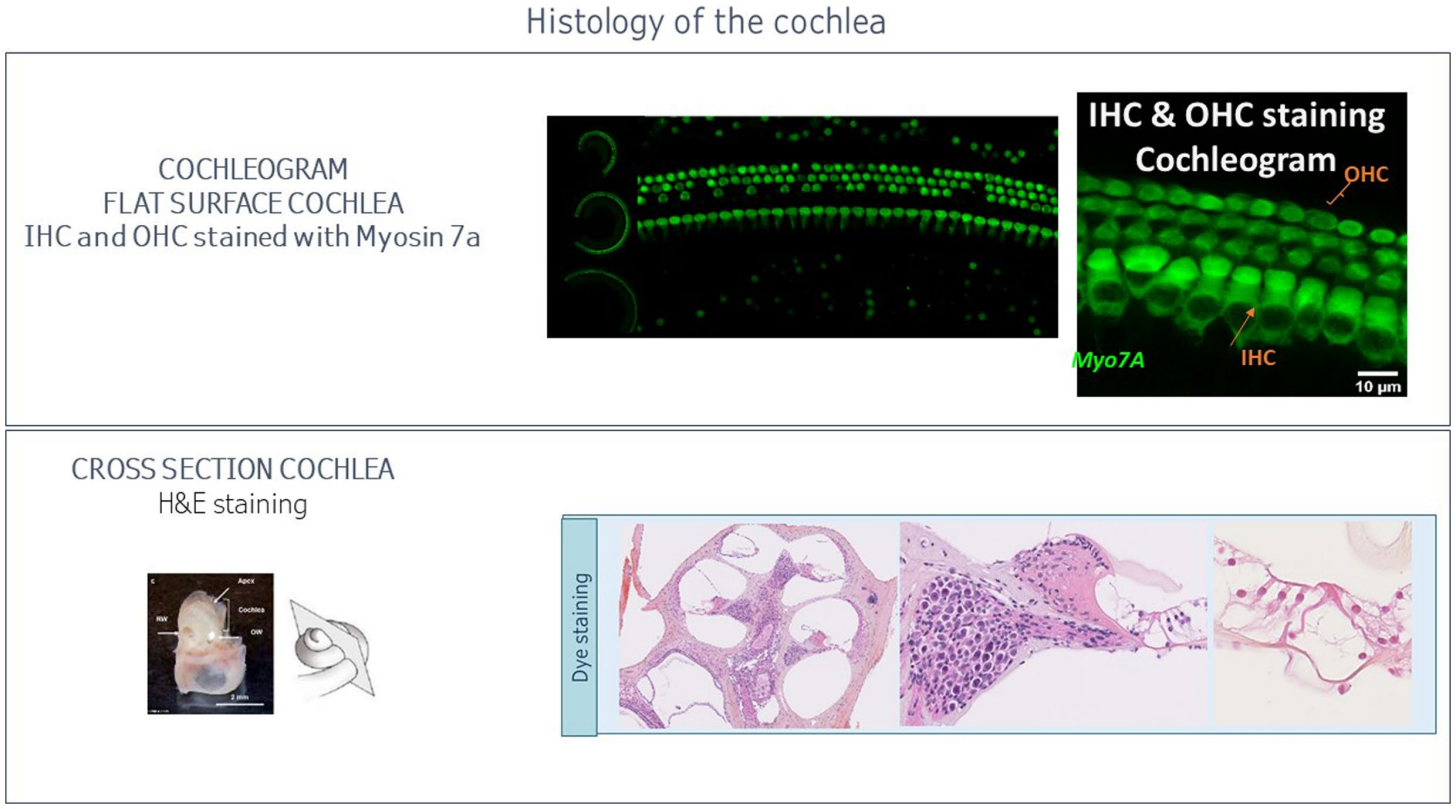
Histology of the cochlea.

The cochleogram is the standard histological procedure for plotting hair cell loss (Viberg and Canlon, 2004; Gauvin et al., 2017) requested by the FDA guideline. Different methods exist for preparing the tissue for counting the hair cells (Neal et al., 2015): sectioning an embedding cochlea (Hirose and Liberman, 2003) and dissecting and staining a whole-mount preparation, to obtain a flat surface preparation (Liberman et al., 2015). This second option requires microdissection skills: after decalcification, the membranous and sensory spiral containing the organ of Corti are dissected out as a flat surface preparation under a dissecting microscope. Whatever the preparation method, the tissues are labeled and then visualized using different microscopic techniques (Figure 3).

Cell counts can be performed manually or automatically with software. The total number of labeled inner and outer hair cells are counted and documented. The cochleogram represents the density of inner and outer hair cells as a function of the distance from apex. The number of inner and outer hair cells is quantified for each level of the cochlea (apical, middle and basal turn) and compared to corresponding ABR threshold and DPOAE amplitude measures. The cochleogram can be performed on the whole cochlea or at pre-defined tonotopic locations (same as for ABR measures).

####### 4.2.2.3.3.2 Histopathology

Evidence of cochlear ototoxicity can be observed in the organ of Corti, the spiral ganglia neurons, the temporal bones, the lateral wall/ stria vascularis. The parameters evaluated to assess ototoxicity include necrosis, cell loss, inflammation and exudate.

Following fixation, auditory tissue samples (middle ear with surrounding bony structures and external auditory canal) are decalcified via immersion in a decal solution. Tissues are then paraffin embedded, sectioned, and stained with H&E (Haemotoxylin and Eosin). Light microscopy is used for histomorphological scoring of parameters: these include inflammatory cell infiltrates, fibrosis, cellular degeneration and necrosis, exudation (in the middle ear), and other parameters as deemed appropriate by the pathologist (Figure 3).

## 5 The clinical phase

### 5.1 Rationale for clinical monitoring

No health authorities have issued guidelines for monitoring ototoxicity in clinical trials of new drug candidates. Hence, with the exception of clinical trials of a potentially ototoxic drug or of an otoprotective agent, ototoxicity monitoring is very rarely included in clinical trials during drug development. In their review of 2021, Coffin et al. indicate that, after consulting with several experts in human ototoxicity monitoring in the U.S. and Europe, none of them were aware of clinical trials collecting audiometric data, as advocated by multiple researchers (Coffin et al., 2021). Nevertheless, some publications address the monitoring of ototoxicity in clinical trial, investigating hearing loss prevention and hearing rehabilitation (King and Brewer, 2018; Le Prell et al., 2023). Nonetheless, some publications address the monitoring of ototoxicity in clinical trial, investigating hearing loss prevention and hearing rehabilitation (King and Brewer, 2018; Lord, 2019; Le Prell et al., 2023). The objective overall of these publications was to identify outcomes and endpoints to be considered for use in the clinical trials.

The objective of an ototoxicity monitoring program is to ensure the early identification of hearing loss and early intervention with the goal of reducing the functional impact of ototoxicity related to treatment. This information can, at times, prevent functional hearing loss by allowing for alternative therapies or by influencing drug prescribing procedures; specifically, smaller or less frequent doses, or interruption or suspension of treatment altogether (King and Brewer, 2018). In the context of a European project called PanCareLIFE, efforts have been made to improve long-term care related to fertility, ototoxicity, and health-related quality of life after cancer in children and adolescents. The study (Strebel et al., 2023) showed that hearing loss and tinnitus are associated with reduced health-related quality of life among childhood cancer survivors— particularly among survivors with both hearing loss and tinnitus. Guideline recommendations should be issued for timely referrals to audiologists for tinnitus symptoms and optimized treatment of hearing loss and tinnitus. Ototoxicity monitoring program should leverage such dramatic situations.

### 5.2 How to monitor

There is no standard protocol for ototoxicity monitoring. The lack of clarity regarding which patients need monitoring, where monitoring should occur, and who should manage ototoxicity in patients reinforces the need for guidelines and protocols. In an UK study (Maru and Malky, 2018), 72% of hearing professionals across the UK indicated that no protocol for ototoxicity management existed within their center.

Encouragingly, three Guidelines on ototoxicity monitoring program have been published: ASHA (American Speech-Language-Hearing Association) in 1994 (Lester et al., 2023), AAA (American Academy of Audiology) in 2009 and HPCSA (Health Professions Council of South Africa) in 2018 (HPCSA, 2018). The two American guidelines provide general recommendations for the monitoring. The first Guidelines developed by the American Speech-Language-Hearing Association (ASHA) (American Speech-Language-Hearing Association, 1994) provide essential recommendations for managing individuals who are receiving drug therapy that has the potential to cause toxic reactions in the inner ear. The recommendations include the following ad verbatim (Konrad-Martin et al., 2018):

- Use a standard definition of an ototoxic hearing shift.
- Conduct pre-treatment counseling regarding potential cochleotoxic effects.
- Include a baseline evaluation preferably before but at least early in treatment.
- Perform monitoring visits at sufficient intervals to document hearing loss progression or fluctuations.
- Perform a post-treatment evaluation followed by longer term monitoring based on the post-treatment outcomes.

In addition to these general recommendations, the ASHA guidelines give specific recommendations on when and how to monitor, taking into account factors such as drug class exposure, patient report, and the ability of the patient to tolerate and accurately perform behavioral testing.

The AAA provides guidance for the implementation of an ototoxicity monitoring program. The guidelines suggest that the audiologist should bear the primary role in the design and development of ototoxicity monitoring programs, including the choice of testing protocols, patient testing or supervision of personnel administering monitoring test(s), interpretation and management of the data derived from such programs, and follow-up management when clinically significant, especially when handicapping degrees of hearing loss are detected. The guidelines also recommend that patients should be counseled before they begin treatment, and a pre-treatment baseline exam is done for accurate interpretation. Careful ototoxicity monitoring can allow the physician to consider altering the treatment regimen before permanent communicative damage occurs or allow the audiologist to work with the patient and their family to maintain communication in those cases, where hearing loss cannot be prevented or reversed (American Academy of Audiology, 2009).

In September 2019, the International Ototoxicity Management Working Group (IOMG) was formed in response to health care gaps in ototoxicity management worldwide at the 9th Biennial Conference of the NCRAR held in Portland, Oregon (Garinis et al., 2021). The IOMG is a global consortium of international stakeholders from universities, task forces, health foundations, professional societies, government agencies and patients created to address healthcare gaps in the clinical management of individuals who experiencing hearing loss, tinnitus, and/or balance difficulties following medical, occupational or environmental exposures to ototoxicants. The IOMG provides guidelines for the clinical management of ototoxicity, including the implementation of ototoxicity monitoring programs. The IOMG’s website provides information on the organization’s leadership and structure, meeting information, and publications.

In January 2020, a subgroup of IOMG committee members volunteered to form the Focus Group on Aminoglycoside Antibiotics (Garinis et al., 2021). The focus group initially met virtually on August 21, 2020, to develop an inventory of barriers and shortcomings of current clinical practices of ototoxicity management in patients receiving aminoglycoside therapies. The outcome of this meeting was to address an immediate need for standardized clinical protocols in patient groups who are routinely treated with aminoglycosides. The IOMG advocates four clinical recommendations for implementing routine and guideline adherent ototoxicity management in patients with cystic fibrosis. These are (a) including questions about hearing, tinnitus, and balance/vestibular problems as part of the routine *CF* case history for all patients; (b) utilizing timely point-of-care measures; (c) establishing a baseline and conducting post-treatment evaluations for each course of intravenous ototoxic drug treatment; and (d) repeating annual hearing and vestibular evaluations for all patients with a history of ototoxic antibiotic exposure.

The testing schedule includes three phases. Baseline measures are performed prior to administration (or as soon as possible after initial dosing) to serve as a reference for detecting significant change (King and Brewer, 2018; Garinis et al., 2021). Immediate post-treatment, test will assess hearing immediately after drug administration (Rizk et al., 2020) Post-Drug Follow-Up test, by regularly monitor patients during and after treatment; will allow to detect any changes in hearing or balance.

Several techniques are at the disposal of the audiologists to assess the hearing functions. Some are designed for early detection of ototoxicity, some for grading ototoxicity, and some for obtaining additional information about ototoxic change and its site of lesion (Campbell, 2018).

According to AAA guidelines, baseline testing should be fairly comprehensive and may include pure tone thresholds in the conventional frequency range, HFA, tympanometry, speech audiometry, and testing of OAEs. After baseline assessment, pure-tone air conduction thresholds (PTA), HFA and OAEs are recommended.

Based on these measures, the audiologist will define the significance and severity of the ototoxicity. The ASHA has defined a significant change in hearing as: “A 20 dB decline in hearing at any single test frequency, or a 10 dB decline at two adjacent frequencies, or loss of response at maximum audiometer outputs for three consecutive frequencies where there was previously measurable hearing. Additionally, these changes need to be confirmed on a follow-up test.”

To classify the grade of ototoxic adverse event, different scales exist (Crundwell et al., 2016), and among them and one of the most widely used, is the National Cancer Institute (NCI) Common Terminology SCALE, including 4 grades, for adults and children.

Test techniques employed and testing schedule may vary according to the drug involved, the patient’s age and ability to perform behavioral testing, and the purpose of the audiologic monitoring.

The use of available clinical guidelines, awareness, communication, and training of the staff should support clinicians in the implementation of ototoxicity monitoring.

## 6 Conclusion

Ototoxicity, leading to hearing loss or balance disorders, is an under diagnosed issue. Lack of monitoring is the primary cause for this. Indeed, unlike eye exams, routine hearing tests are not a standard health care practice. The symptoms are variable, often subtle, and frequently attributed to other causes. Further, ototoxicity may be delayed, occur gradually over time, or cumulatively, making it challenging to associate the symptoms with a specific cause. The lack of ototoxicity awareness and monitoring leads to misdiagnosis or delayed recognition.

There are profound health risks caused by ototoxicity. It is crucial for healthcare providers to be aware of the potential risks associated with certain medications and treatments. Regular audiologic monitoring, patient education, and communication between healthcare providers can help identify ototoxicity early, allowing for appropriate intervention and management.

Ototoxicity research and surveillance in drug development will contribute to better understanding and prevention. Preventive measures for hearing loss are cost-effective and can bring great benefit to individuals and communities. However, patients may not always report subtle changes in their hearing or balance or consider the cause to be the medications they are receiving. Lack of communication hinders ototoxicity identification. This leads to most of the data regarding ototoxic effects of drugs coming from post-marketing reports when potentially irreversible hearing damage has already occurred.

When compared with other targeted sensory indications, such as ophthalmology, there is a clear lack of regulatory guidance on ototoxicity. There are few resources to ensure drugs and treatments with potential ototoxic effects are thoroughly evaluated for safety during the approval process. It is imperative to use a comprehensive drug safety approach that includes monitoring for potential adverse effects on hearing and balance.

Nonclinical testing for auditory safety allows the assessment of potential ototoxicity properties earlier in the drug development process, thus reducing cost, and avoiding potential clinical risks. Development of novel drugs should include auditory safety assessments, while greater efforts are needed to prevent ototoxicity caused by existing treatments.

## Author contributions

M-PP: Writing – original draft, Writing – review & editing. LB: Writing – review & editing. SM: Writing – review & editing. SP: Writing – review & editing. MP: Writing – review & editing. HL: Writing – review & editing. DH: Writing – review & editing. AT: Writing – review & editing. GN: Writing – review & editing.
